# RETRACTION: Comprehensive Assessment of Risk Factors and Pathogenic Characteristics of Wound Infections Following Thoracoscopic Radical Resection for Lung Cancer

**DOI:** 10.1111/iwj.70405

**Published:** 2025-03-21

## Abstract

**RETRACTION:** XuJ. and ZhuJ. , “Comprehensive Assessment of Risk Factors and Pathogenic Characteristics of Wound Infections Following Thoracoscopic Radical Resection for Lung Cancer,” International Wound Journal 21, no. 4 (2024): e14830, 10.1111/iwj.14830.38531534 PMC10965314

The above article, published online on 26 March 2024, in Wiley Online Library (http://onlinelibrary.wiley.com/), has been retracted by agreement between the journal Editor in Chief, Professor Keith Harding; and John Wiley & Sons Ltd. Following an investigation by the publisher, all parties have concluded that this article was accepted solely on the basis of a compromised peer review process. The editors have therefore decided to retract the article. The authors did not respond to our notice regarding the retraction.

**RETRACTION:** J. Xu and J. Zhu , “Comprehensive Assessment of Risk Factors and Pathogenic Characteristics of Wound Infections Following Thoracoscopic Radical Resection for Lung Cancer,” International Wound Journal 21, no. 4 (2024): e14830, 10.1111/iwj.14830.38531534 PMC10965314

The above article, published online on 26 March 2024, in Wiley Online Library (http://onlinelibrary.wiley.com/), has been retracted by agreement between the journal Editor in Chief, Professor Keith Harding; and John Wiley & Sons Ltd. Following an investigation by the publisher, all parties have concluded that this article was accepted solely on the basis of a compromised peer review process. The editors have therefore decided to retract the article. The authors did not respond to our notice regarding the retraction.

